# Octreotide-mediated neurofunctional recovery in rats following traumatic brain injury. Role of H_2_S, Nrf2 and TNF-α

**DOI:** 10.1590/ACB361204

**Published:** 2022-02-23

**Authors:** Jie Zhou, Li Cao, Xia Feng, Baosheng Zhou, Linshan Li

**Affiliations:** 1 BM. Department of Neurosurgery - General Hospital of TISCO – Taiyuan - Shanxi, China.; 2 BM. Department of Neurosurgery - The 940th Hospital of Joint Logistics Support Force of PLA - Lanzhou - Gansu, China.; 3 BM. Department of Neurosurgery - Tianjin First Central Hospital - Tianjin, China; 4 BM. Department of Neurosurgery - Tianjin First Central Hospital - Tianjin, China.; 5 BM. Department of Neurosurgery - Shuangqiao Economic and Technological Development Zone People’s Hospital - Chongqing, China.

**Keywords:** Octreotide, Trauma, Morris Water Maze Test, Prefrontal Cortex, Cystathionine-γ-Lyase, Rats

## Abstract

**Purpose:**

To explore the role and mechanisms of octreotide in neurofunctional recovery in the traumatic brain injury (TBI) model.

**Methods:**

Rats were subjected to midline incision followed by TBI in the prefrontal cortex region. After 72 hours, the behavioural and neurological deficits tests were performed, which included memory testing on Morris water maze for 5 days. Octreotide (15 and 30 mg/kg *i.p.*) was administered 30 minutes before subjecting to TBI, and its administration was continued for three days.

**Results:**

In TBI-subjected rats, administration of octreotide restored on day 4 escape latency time (ELT) and increased the time spent in the target quadrant (TSTQ) on day 5, suggesting the improvement in learning and memory. It also increased the expression of H_2_S, Nrf2, and cystathionine-γ-lyase (CSE) in the prefrontal cortex, without any significant effect on cystathionine-β-synthase. Octreotide also decreased the TNF-α levels and neurological severity score. However, co-administration of CSE inhibitor (D,L-propargylglycine) abolished octreotide-mediated neurofunctional recovery, decreased the levels of H_2_S and Nrf2 and increased the levels of TNF-α.

**Conclusions:**

Octreotide improved the neurological functions in TBI-subjected rats, which may be due to up-regulation of H_2_S biosynthetic enzyme (CSE), levels of H_2_S and Nrf2 and down-regulation of neuroinflammation.

## Introduction

Traumatic brain injury to the brain results from an external mechanical force that may lead to temporary or permanent impairment^1^. It continues to affect millions of individuals around the world every year, and its manifestations may vary depending on the severity of the traumatic impact. The incidences of traumatic brain injury are more often found in a very young age-group (0–4 y), adolescents and young adults (15–24 y) and elderly (>65 y). With the significant improvement in the medical sciences, the rate of death arising due to traumatic brain injury is significantly reduced. However, there is a growing population of patients living with significant disabilities as a result of brain injury^2^. Therefore, there is a need to identify new drugs or interventions that may promote healing and improve disabilities after traumatic brain injury.

Octreotide is a long-acting somatostatin analogue and a synthetic cyclic peptide consisting of eight aminoacids. It is employed to manage acromegaly, diarrhoea in patients with vasoactive intestinal peptide-secreting tumours, acute haemorrhage from oesophageal varices in liver cirrhosis, fistula by reducing gastrointestinal fistula and refractory hypoglycemia^3^. Apart from that, it has been shown to produce beneficial effects in brain-related problems, including idiopathic intracranial hypertension^4^, nociception^5^ and status epilepticus^6^, as well as to exert protection in ischemic stroke^7^ and severe acute pancreatitis-induced brain damage^8^. Accordingly, it was hypothesized that octreotide may exert beneficial effects in traumatic brain injury-subjected rats.

Neuroinflammation is a key event in traumatic brain injury-induced deleterious effects, and increase in the TNF-α levels is widely employed as a marker of neuroinflammation^9^^,^ 10. Along with the onset of neuroinflammation, there is also an imbalance in oxidative stress and endogenous antioxidants. Indeed, there is a significant decline in the levels of Nrf2, a transcriptional regulator of endogenous antioxidants, during traumatic brain injury^11^^,^ 12. There are studies suggesting that octreotide may produce beneficial effects in ischemic stroke by up-regulating the transcription factor Nrf2 and down-regulating the NF-κB expression^7^. Hydrogen sulfide (H_2_S) is a gaseous neurotransmitter that has been synthesized endogenously by cystathionine-γ-lyase and cystathionine-β-synthase^13^. Studies have shown the function of physiological and pathophysiological roles of H_2_S in the brain including traumatic brain injury^14^^,^ 15. Furthermore, other studies have pointed out the close interrelationship between H_2_S, neuroinflammation and Nrf2^16^^,^ 17.

Accordingly, it was hypothesized to explore the role of neuroinflammation, Nrf2, H_2_S and its biosynthetic enzymes in octreotide-mediated beneficial effects in traumatic brain injury model.

## Methods

In the present study, Wistar albino rats were employed. The experimental protocol was approved by Taiyuan Iron and Steel Group, general hospital, by the ethic no. 202078954e. Octreotide and DL-propargylglycine were procured from Sigma-Aldrich (San Luis, MO, United States). The enzyme-linked immunosorbent assay (ELISA) kits for the quantitative estimation of Nrf2, cystathionine-γ-lyase and TNF-α were obtained from Lifespan Biosciences (Seattle, WA, United States). The fluorometric assay kit of cystathionine-β-synthase was produced by BioVision (Milpitas, CA, United States). The doses of octreotide^18^^,^ 19 and DL-propargylglycine^20^ were selected based on literature.

### Traumatic brain injury model

Animals were anesthetized using 4% isoflurane, and a midline incision was made to expose the skull. Using stereotactic apparatus, the prefrontal cortex region was identified (at coordinates P = -2 and L = 1.4). Traumatic brain injury (TBI) was induced in the prefrontal cortex region using a pneumatic piston (calibrated at 40 psi of pressure and depth of 6 mm). Thereafter, the animals were allowed to recover. In sham, all procedures were performed, except inducing injury^21^.

### Behavioural tests

To assess the functionality of the nervous system, behavioural tests were performed after 72 hours of TBI.

### Neurological deficits scoring

Animals were assessed through neurological severity tests and assigned scores from 0 (normal) to 10 (the most severe form), based on total neurological deficits^22^^,^ 23.

### Memory testing on Morris water maze

After three days of TBI, *i.e.*, on the 4^th^ day after brain injury, animals were subjected to memory testing on the Morris water maze. The animals were subjected to acquisition trials on four days (4^th^, 5^th^, 6^th^ and 7^th^ day after injury), and it was followed by a test for the retrieval of memory on the 8^th^ day after injury. The escape latency time (ELT) measured on the first four days (acquisition trials) served as an index of learning. The time spent in the target quadrant (TSTQ) on the 8^th^ day after the injury was depicted as the index of retrieval (index of memory or retention)^24^.

### Determination of biochemical parameters

After the determination of ELT and TSTQ on the Morris water maze test, rats were sacrificed followed by the isolation of the brain. Afterwards, the brain was homogenized in a phosphate buffer solution, and the supernatant of the homogenate brain was used for the quantification of different biochemical parameters, including H_2_S, CSE, CBS, TNF-α and Nrf2.

### H_2_S determination

The H_2_S levels in the brain homogenate were measured using monobromobimane (MBB) method coupled with reversed-phase high-performance liquid chromatography (RP-HPLC). In this method, derivatization of hydrogen sulfide with excess MBB at room temperature to form sulfide dibimane (SDB) and the fluorescence were analysed by RP-HPLC using fluorescence detectors^25^.

### Quantification of cystathionine-β-synthase, cystathionine-γ-lyase, TNF-α And Nrf2

The expression of cystathionine-β-synthase (CBS) was determined in the brain homogenate using fluorometric activity assay kits. The expression of cystathionine-γ-lyase (CSE), TNF-α and Nrf2 was determined in the brain supernatants using commercially available ELISA kit as per manufacture instructions.

### Experimental protocol

Six groups were employed, and each one was comprised of eight animals:

1. Sham: Under anaesthesia, a midline incision was made in the skull without induction of injury. After 72 hours, the behavioural and neurological deficits tests were performed. Afterwards, animals were subjected to memory testing on the Morris water maze for five days (4^th^, 5^th^, 6^th^, 7^th^ day and 8^th^ day). During five days of testing, ELT (4^th^, 5^th^, 6^th^, 7^th^ day) and TSTQ (8^th^ day) were measured in the Morris water maze test. On the last day, the brain was removed after killing the animals and homogenized for measuring biochemical parameters;
2. TBI: TBI was induced in the prefrontal cortex region of a rat by using a pneumatic piston. After 72 hours, the behavioural and neurological deficits tests were performed. Afterwards, ELT, TSTQ and biochemical parameters were measured as described in the sham control group;
3. Octreotide (15 mg/kg *i.p.*) in TBI: Octreotide (15 mg/kg *i.p.*)was administered 30 minutes before subjecting the rats to TBI, and its administration was continued for three days after TBI. The rest of the protocol was the same as per the sham control group;
4. Octreotide (30 mg/kg *i.p.*) in TBI: Octreotide (30 mg/kg *i.p.*) was administered 30 minutes before subjecting to TBI and its administration was continued for three days after TBI. The rest of the protocol was the same as per the sham control group;
5. D,L-propargylglycine (25 mg/kg i.p.) and octreotide (30mg/kg) in TBI: D,L-propargylglycine (25 mg/kg *i.p.*)was co-administered with octreotide 30 minutes before subjecting the rats to TBI, and it was continued for three days after TBI. The rest of the protocol was the same as per the sham control group;
6. D,L-propargylglycine (50 mg/kg i.p) and octreotide (30 mg/kg) in TBI: D,L-propargylglycine (50 mg/kg *i.p.*) was co-administered with octreotide (30 mg/kg) 0.5 hour before subjecting the rats to TBI, and their administration was continued for three days after TBI. The rest of the protocol was the same as per the sham control group.

## Results

### Traumatic brain injury produced neurofunctional deficits and impairment in learning and memory

**Figure 1:**
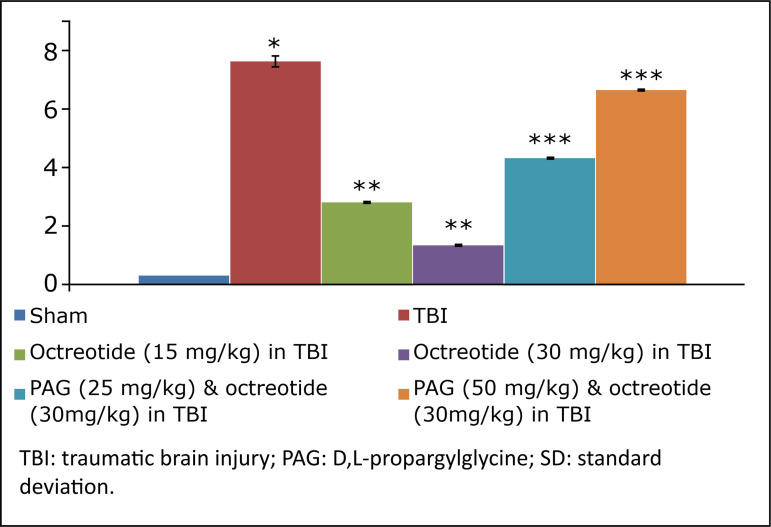
The results of neurological severity score due to TBI in different experimental groups. Values are given in mean ± SD. *P<0.05 *vs*. sham; **P<0.05 *vs*. TBI; ***P<0.05 *vs*. octreotide (30 mg/kg) in TBI.

Rats were subjected to midline incision followed by TBI in the prefrontal cortex region using a stereotaxic apparatus. The TBI-subjected rats developed significant neurofunctional impairment in comparison to the sham group. There were significant behavioural alterations in terms of increase in neurological severity score (Fig. 1) assessed on the 3^rd^ day following TBI in rats.

**Figure 2:**
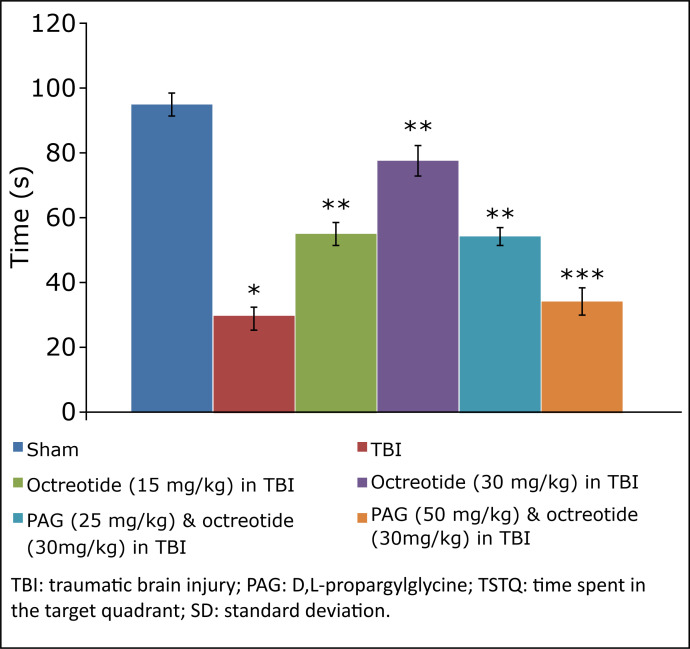
Effect of different interventions on TSTQ measured on 8^th^ day of Morris water maze test. Values are given in mean ± SD. *P<0.05 *vs*. sham; **P<0.05 *vs*. TBI; ***P<0.05 *vs*. octreotide (30 mg/kg) in TBI.

The assessment of learning and memory in terms of ELT and TSTQ in TBI-subjected animals started on the 4^th^ day on the Morris water maze test and continued for five days (four days of acquisition and one day of retrieval). In brain injury-subjected rats, there was a deficit in the learning, as depicted by a no significant change in the ELT on the 7^th^ day (fourth day of acquisition trial) in comparison to the 4^th^ day (first day of trial) (Table 1). Furthermore, the parameter to assess the memory, *i.e.*, TSTQ, was significantly lower on the 8^th^ day in brain injury-subjected rats in comparison to the sham group, suggesting a significant impairment in memory in injury-subjected rats (Fig. 2).

**Table 1:** Effect of different interventions on escape latency time (ELT) on Morris water maze test. *p< 0.05 *vs*. day 1 ELT of sham; **p< 0.05 *vs*. day 4 ELT of sham; ***p< 0.05 *vs*. day 4 ELT of TBI; ****p< 0.05 *vs*. day 4 ELT of octreotide (30 mg/kg) in TBI.

**S. No**	**Groups**	**Day I ELT(s)**	**Day 4 ELT(s)**
1	Sham	97.6 ± 6.8	32.6 ± 3.4^*^
2	TBI	106.2 ± 6.1	89.2 ± 5.1^**^
3	Octreotide (15 mg/kg) in TBI	103.1 ± 5.8	65.2 ± 4.3^***^
4	Octreotide (30 mg/kg) in TBI	100.4 ± 4.0	42.3 ± 6.3^***^
5	D,L-propargylglycine (25 mg/kg) and octreotide (30 mg/kg) in TBI	102.8 ± 6.2	48.7 ± 5.1^****^
6	D,L-propargylglycine (50 mg/kg) and octreotide (30 mg/kg) in TBI	104.6 ± 3.7	72.2 ± 5.3^****^

TBI: traumatic brain injury.

### Effect of octreotide on the neurofunctional and cognitive deficit in injury-subjected rats

Octreotide (15 and 30 mg/kg *i.p.*) was administered 30 minutes before subjecting the rats to TBI, and its administration was continued for three days after TBI. Administration of octreotide significantly attenuated brain injury-induced behavioural deficits in terms of decrease in neurological severity score (Fig. 1) assessed on the 3^rd^ day following TBI. Moreover, there was a significant decrease in ELT on the 7^th^ day (4^th^ day of acquisition trial) in comparison to ELT on the 4^th^ day (1^st^ day of trial) (Table 1), suggesting the improvement in learning. The value of TSTQ was also significantly increased on the 8^th^ day in octreotide-treated rats (Fig. 2), suggesting the memory improvement.

### Effect of octreotide on biochemical parameters in brain injury-subjected rats

**Figure 3:**
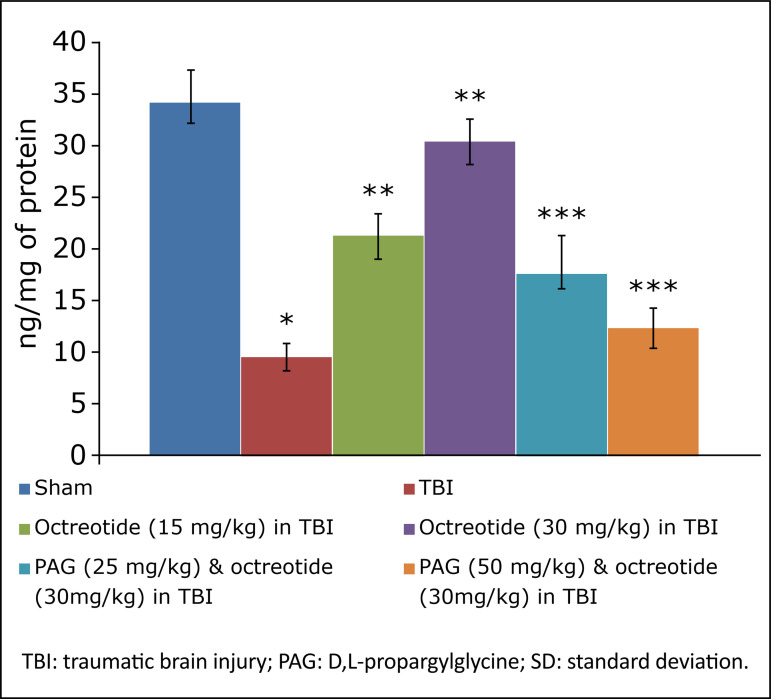
Effects of different interventions on the hydrogen sulfide levels in the brain due to TBI in different experimental groups. Values are given in mean ± SD. *P<0.05 *vs*. sham; **P<0.05 *vs*. TBI; ***P<0.05 *vs*. octreotide (30 mg/kg) in TBI.

**Figure 4:**
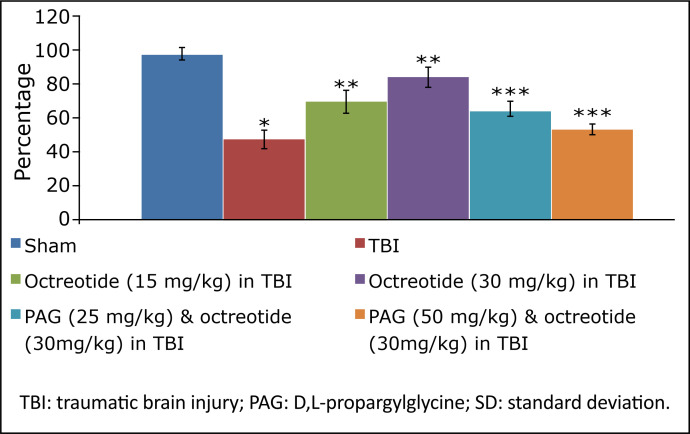
Effects of different interventions on the cystathionine-γ-lyase in the brain due to TBI in different experimental groups. Values are given in mean ± SD. *P<0.05 *vs*. sham; **P<0.05 *vs*. TBI; ***P<0.05 *vs*. octreotide (30 mg/kg) in TBI.

**Figure 5:**
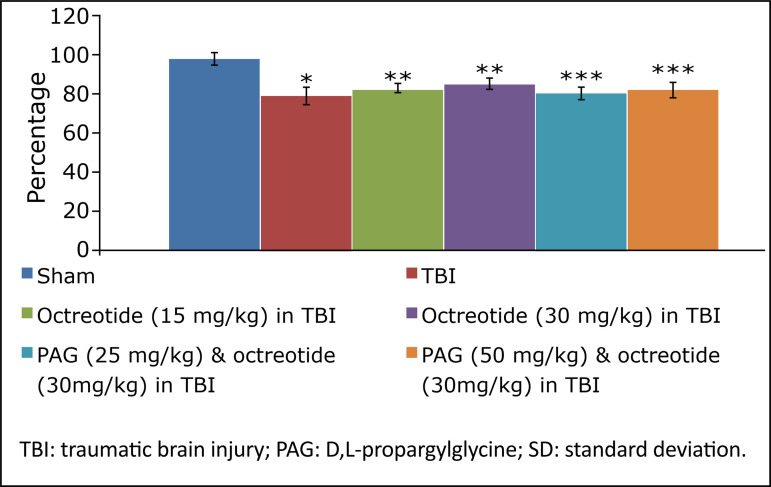
Effects of different interventions on the cystathionine-β-synthase in the brain due to TBI in different experimental groups. Values are given in mean ± SD. *P<0.05 *vs*. sham; **P<0.05 *vs*. TBI; ***P<0.05 *vs*. octreotide (30 mg/kg) in TBI.

**Figure 6:**
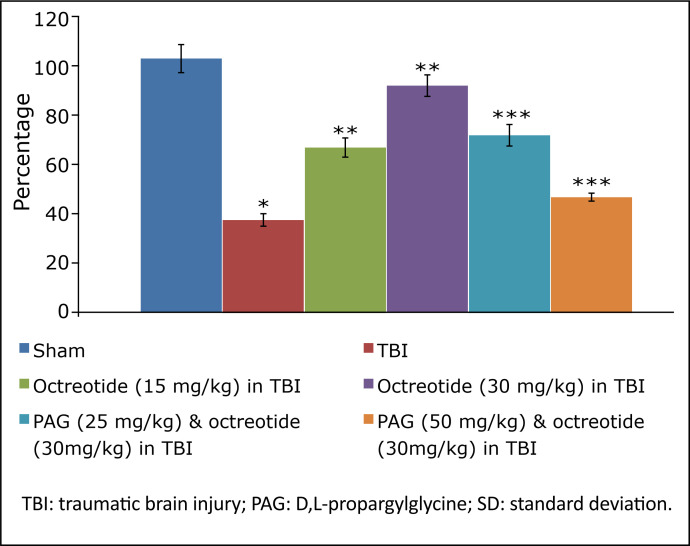
Effect of different interventions on the Nrf2 levels measured in prefrontal cortex region of brain. Values are given in mean ± SD. *P<0.05 *vs.* sham; **P<0.05 *vs*. TBI; ***P<0.05 *vs*. octreotide (30 mg/kg) in TBI.

**Figure 7:**
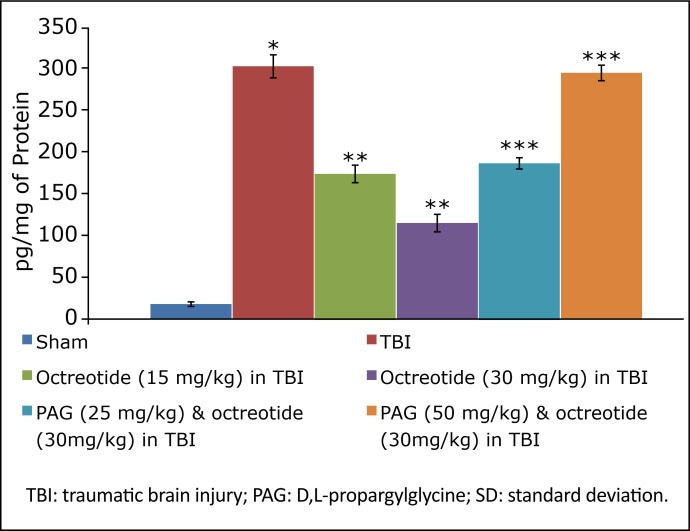
Effect of different interventions on the TNF-α levels measured in prefrontal cortex region of brain. Values are given in mean ± SD. *P<0.05 *vs*. sham; **P<0.05 *vs.* TBI; ***P<0.05 *vs*. octreotide (30 mg/kg) in TBI.

TBI led to a significant decrease in the levels of H_2_S (Fig. 3), CSE (Fig. 4), and with no significant change in CBS expression (Fig. 5). There were significant decrease in the Nrf2 (Fig. 6) and increase in the TNF-α levels (Fig. 7) in TBI-subjected rats. Administration of octreotide in brain injury-subjected rats exhibited significant rise in levels of H_2_S (Fig. 3) along with increase in the activity of CSE, an enzyme responsible for the synthesis of H_2_S (Fig. 4). However, administration of octreotide did not exhibit significant effect on the activity of another H_2_S biosynthesizing enzyme, CBS (Fig. 5). Moreover, octreotide restored the expression of Nrf2 (Fig. 6) and decreased neuroinflammation as assessed by a decrease in the TNF-α levels in TBI-subjected animals.

### Effect of D,L-propargylglycine on octreotide-induced improvement in neurofunctional deficits and biochemical changes in traumatic brain injury-subjected rats

Co-administration of CSE inhibitor, D,L-propargylglycine (25 and 50 mg/kg *i.p.*) significantly attenuated the octreotide-mediated decrease in the neurological severity score (Fig. 1). It also abolished octreotide-mediated improvement in learning (Table 1) and memory (Fig. 2) in the Morris water maze test. Furthermore, it also diminished the effects of octreotide on the H_2_S levels and CSE activity as depicted by a decrease in levels of H_2_S (Fig. 3) and CSE expression (Fig. 4). However, it did not modulate the activity of CBS in TBI-subjected rats (Fig. 5), but extinguished the octreotide-induced increase in the expression of Nrf2 (Fig. 6) and decrease in the TNF-α levels (Fig. 7) in response to TBI.

## Discussion

Trauma is one of the common causes of brain injury^26^, and there have been different models employed by scientists to identify the pharmacological agents to mitigate the deleterious effects associated with the TBI^27^^,^ 28. In this study, traumatic injury to the brain led to significant neurofunctional impairment. There was a significant rise in the neurological severity score, assessed three days after the injury. Moreover, there was a significant impairment in the learning (increase in ELT) and memory (decrease in TSTQ) in TBI-subjected rats. Previous studies have been shown that there is impairment in behavioural functions in animals subjected to TBI^29^^-^ 31.

In this study, treatment with octreotide ameliorated TBI-induced increase in neurological severity score and decrease in cognitive functions. There was a significant decrease in the day 4 ELT and day 5 TSTQ in octreotide-treated rats. Octreotide is a somatostatin analogue, and, apart from its hormonal functions, studies have shown its widespread potential in ameliorating the pathophysiological state of different diseases^32^, including neuroendocrine gastrointestinal tumors^33^ and pancreatic tumors^34^, cardiovascular disease^35^^,^ 36 and Crohn’s disease^37^. However, to the best of our knowledge, it is the first study showing the potential of octreotide in improving neurofunctional aspects of TBI-subjected rats.

In the present investigation, treatment with octreotide also ameliorated TBI-induced biochemical alterations. In TBI-subjected rats, there was a significant decline in the expression of H_2_S along with the CSE activity, one of the enzymes involved in H_2_S synthesis. Interestingly, there was no significant change in the activity of another H_2_S synthesis, *i.e.*, CBS. Accordingly, TBI may alter the activity of CSE, which might lead to a decrease in the H_2_S levels in the brain. Earlier studies have shown that the decrease in the H_2_S levels may be deleterious to the brain^38^^,^ 39. However, treatment with octreotide restored the CSE activity and increased the H_2_S levels, suggesting that octreotide-mediated increase in the H_2_S levels may contribute to improve neurofunctional aspects in TBI-subjected rats. The role of H_2_S in octreotide-mediated beneficial effects was further supported as the administration of D,L-propargylglycine(CSE inhibitor) abolished the effects of octreotide. Along with it, there was also a decrease in the H_2_S levels and CSE activity in D, L-propargylglycine administered rats.

In this study, treatment with octreotide also abolished TBI-induced increase in neuroinflammatory marker (TNF-α levels) and Nrf2 levels. It suggests that octreotide-mediated decrease in neuroinflammation and increase in endogenous antioxidant actions may contribute to improve neuronal functions in TBI-subjected rats. Moreover, D,L-propargylglycineattenuated the effects of octreotide on neuroinflammation and Nrf2. It suggests that octreotide may regulate the expression of CSE, which may increase the H_2_S levels to decrease neuroinflammation and increase endogenous antioxidant actions to confer neurofunctional protection against TBI in rats.

## Conclusion

It is concluded that octreotide improves TBI and neurological function in the brain, possibly through up-regulation of H_2_S, Nrf2, and CSE in the prefrontal cortex and down-regulation TNF-α level and neuroinflammation.
